# Early retinal differentiation of human pluripotent stem cells in microwell suspension cultures

**DOI:** 10.1007/s10529-016-2244-7

**Published:** 2016-11-03

**Authors:** Vishal S. Sharma, Rana Khalife, Rui Tostoes, Leonard Leung, Rose Kinsella, Ludmilla Ruban, Farlan S. Veraitch

**Affiliations:** Department of Biochemical Engineering, University College London, Gower Street, London, WC1E 6BT UK

**Keywords:** Bioreactor, Embryoid body, Induced pluripotent stem cells, Matrigel, Microwell, Orbital shaking culture, Retinal differentiation

## Abstract

**Objective:**

To develop a microwell suspension platform for the adaption of attached stem cell differentiation protocols into mixed suspension culture.

**Results:**

We adapted an adherent protocol for the retinal differentiation of human induced pluripotent stem cells (hiPSCs) using a two-step protocol. Establishing the optimum embryoid body (EB) starting size and shaking speed resulted in the translation of the original adherent process into suspension culture. Embryoid bodies expanded in size as the culture progressed resulting in the expression of characteristic markers of early (Rx, Six and Otx2) and late (Crx, Nrl and Rhodopsin) retinal differentiation. The new process also eliminated the use of matrigel, an animal-derived extracellular matrix coating.

**Conclusions:**

Shaking microwells offer a fast and cost-effective method for proof-of-concept studies to establish whether pluripotent stem cell differentiation processes can be translated into mixed suspension culture.

**Electronic supplementary material:**

The online version of this article (doi:10.1007/s10529-016-2244-7) contains supplementary material, which is available to authorized users.

## Introduction

Human induced pluripotent stem cells (hiPSC) represent an unlimited supply of differentiated tissue-specific cells from any individual. Generating these cells requires improved differentiation processes, which can be complex, difficult to scale, highly variable and driven by the use of expensive growth factors. Currently, the majority of differentiation protocols use adherent two-dimensional planar culture which are commonly initiated by the formation of cellular aggregates known as embryoid bodies (EBs) which are plated onto T-flask and well plate formats. Adherent culture of EBs is resource intensive and less scalable than rotary or orbital shaken suspension cultures (Carpenedo et al. 2007). Mixed suspension cultures have been shown to improve the yield of EB processes (Yirme et al. 2008) and EB size plays a crucial role in defining lineage commitment (Hwang et al. 2009).

Moving to well mixed suspension cultures can offer a range of benefits ranging from more homogenous microenvironments to less intensive use of expensive soluble factors and access to a host of established bioreactor technologies. Despite the wide-ranging of benefits associated with suspension culture of EBs the majority of stem cell labs persist with traditional 2D planar culture formats. This can, in part, be attributed to the number of cells, extensive development man-hours and specialised bioreactor equipment which are seen by many as a barrier to entry by many cell biology laboratories.

Shaking cultures in microwells has been developed as a scale-down mimic of large-scale cultures for process development studies (Silk et al. 2010). Interestingly, this technology offers a scalable, small-scale suspension format which can be used to quickly assess whether two-dimensional planar or three-dimensional static differentiation processes can be successfully adapted to suspension culture. The technology is cheap and utilises standard laboratory incubators, microwell plates and shaking platforms. The goal of this study was to investigate whether a typical lab-scale hiPSC differentiation protocol could be quickly adapted to microwell suspension culture.

For this study, we focussed on the retinal differentiation of hiPSC, a major target for pluripotent stem cells. The generation of retinal neurons such as photoreceptors holds great promise for the treatment of photoreceptor dystrophies and the study of disease mechanisms. The published differentiation protocols (for review see Ramsden et al. 2014) typically rely upon the formation of cellular aggregates known as EBs followed by exposure to a cocktail of expensive growth factors in two-dimensional planar or three-dimensional static culture. The ability to translate this process into 3D orbital shaken suspension culture was undertaken in a simple two-step adaption using the 24-well microwell platform. First we assessed the optimal starting size for EBs which was controlled using forced aggregation (Karp et al. 2007) before establishing an optimum shaking speed for the maintenance of intact EBs. Gene expression analyses revealed retinal differentiation was permissible in orbital shaking suspension cultures using readily available technologies which can be easily adopted in basic stem cell biology laboratories without the need to invest in expensive small scale bioreactor platforms.

## Materials and methods

### Human induced pluripotent stem cells (hiPSC) culture

MSU001 Human induced pluripotent stem cells (hiPSC) cell line used in these experiments, (kindly donated by Jose Cibelli, Michigan State University) were originally derived from human adult dermal fibroblasts using retroviral transduction previously described by Takahashi et al. (2007).

hiPSC cells were routinely co-cultured on mytomycin C (Sigma-Aldrich) inactivated mouse embryonic fibroblasts in culture medium containing KnockOut Dulbecco’s modified Eagle’s medium (KnockOut DMEM), supplemented with 20% knockout serum replacement (KOSR), 1% minimum essential medium non-essential amino acids (MEM NEAA), 2 mM l-Glutamine, 0.1 mM ß-mercaptoethanol (all Invitrogen), and 10 ng basic fibroblastic growth factor (bFGF)/ml (R&D Systems). The medium was replaced daily except the day after each passage. hiPSC cells were used between passage 56 and 80. hiPSC cells were passaged twice per week at a ratio of 1:2 or 1:3 on reaching 70–80% confluence.

### Scraped embryoid bodies (EBs) formation

For manually scraped embryoid bodies (EBs), hiPSC were harvested from T25 flasks at 70–80% confluency, by manual scraping. Prior to detachment the spent medium was removed and cells were incubated for 30 min with 3 ml EB formation medium which consisted of knockout-DMEM, 10% knockout serum-replacement (KOSR), 2 mM l-glutamine, 2 mM non-essential amino acids supplemented with 1 ng dickkopf-related protein-1 (dkk1)/ml (Cambridge Biosciences), 1 ng Noggin/ml, 5 ng insulin-like growth factor (IGF-1)/ml (both from R&D Systems). Aggregates were formed by scraping a Fine Tip Mini Pastette (Alpha Laboratories) across the growth area. These were pelleted and resuspended in retinal induction medium (DMEM/F12 (Invitrogen), supplemented with 1 ng Noggin/ml, 5 ng IGF-1/ml, 1 ng dkk1/ml, 10% (w/v) KOSR, 1% chemically-defined, serum-free supplement based on Bottenstein’s N-1 formulation (N2) supplement (Invitrogen), and 2 mM l-glutamine (Lamba et al. 2006). Suspended aggregates were transferred to low attachment bacterial-grade culture dishes (Sterilin, 3 cm diam.) and incubated at 37 °C with 5% CO_2_ for 3 days; cultures were manually agitated once daily to avoid mass clumping.

### Forced aggregation

Uniform sized stem cell aggregates (1000 cells, 5000 and 10,000 cells/EB) were formed using Aggrewell plates (Stemcell Technologies) in accordance with the manufacturer’s instructions in retinal induction medium supplemented with 50 mM Y-27,632 ROCK inhibitor (ROCKi) (Millipore). After 24 h harvested aggregates were transferred to a 10 ml centrifuge tube and allowed to settle before the retinal induction medium containing ROCKi was replaced with standard retinal induction medium (Lamba et al. 2006). EBs were transferred to low attachment, 3 cm bacterial grade dishes and returned to an incubator at 37 °C for two additional days.

### Orbital shaking of EB culture

Three days after the initiation of forced aggregation, EBs were transferring to a 10 ml centrifuge tube and allowed to settle for 3 min. Spent retinal induction medium was aspirated and replaced with phase 2 (P2) retinal differentiation medium (Osakada et al. 2009) which consisted of DMEM/F12 (Invitrogen), supplemented with 10 ng Noggin/ml, 10 ng IGF-1/ml (Miltenyi Biotec), 10 ng dkk1/ml, 1% N2 supplement (Invitrogen), 5 ng/ml serum-free supplement for neural cell culture (B27) supplement (Invitrogen), 5 ng bFGF/ml, and 1% l-glutamine.

EBs were mixed and aspirated using wide tip plastic Pasteur pipettes (Starlabs) and 30–40 EBs were transferred to each well of ultra-low attachment 24-well culture plates (Corning). The well dimensions were: ht = 17.4 mm, top internal diam. = 16.3 mm and bottom internal diam. = 15.6 mm. Additional P2 medium was added to make 500 μl per well. No Matrigel was added. Culture plates were shaken with orbital diam. 10 mm set at 0.08 g at 37 °C for the remainder of the differentiation with ~90% medium changes every 2 days.

### Retinal differentiation

Three days after the initiation of forced aggregation EBs were harvested by aspiration with 5 ml pipettes and collected in 10 ml centrifuge tubes for settling.

Spent medium was replaced with phase 2 (P2) retinal differentiation medium. The aggregates were finally transferred to either Matrigel coated standard 24-well plates (for adherent culture) or ultra-low adhesion standard 24-well plates at 30–40 EBs per well before being placed on a shaker at 37 °C, 5% CO_2_. Medium was replaced every 2 days until day 15 when cultures were harvested for analysis assays.

### EB size calculation in shake cultures

Variation of EB size over time was assessed by calculating the average sizes of EBs (n = 100) formed by scraping or forced aggregation. Periodic light microscopy micrographs were used to take three measurements of diam. (horizontal, vertical and diagonal). Pixel lengths were measured and converted to μm using Image J software after 1, 3, 7, 10 and 15 days. EB size was calculated as the mean three diam.s and the data was grouped into frequency plots after being counted and categorised into size brackets from 0 to 1200 μm in 50 μm increments.

### EB cryosectioning

EBs harvested for cryosectioning were incubated in 4% (w/v) paraformaldehyde (PFA) for 20 min at room temperature. PFA was replaced with 30% sucrose/phosphate-buffered saline (PBS) buffer and re-incubated overnight at 4 °C. Dehydrated EBs were transferred to cryosection moulds and excess sucrose solution removed before embedding medium OCT (Tissue-Tek) was added to cover the aggregates and fill the moulds. OCT filled moulds were frozen at −80 °C and later sectioned using a Thermo Scientific Cryotome FSE Cryostat into 10 μm slices. Slices were picked using Microslide Superfrost Plus White (VWR) glass slides, dried at room temperature for at least 1 h before being stored at −80 °C. Sections removed from frozen storage were acclimatised to room temperature before drying for up to 2 h prior to commencing immunohistochemical analyses.

### Immunostaining

Adherent cultures or cyrosection slides were fixed in 4% (w/v) PFA for 20 min at room temperature. The PFA was removed and cells were washed with PBS before being treated with blocking buffer (0.3% Triton and 5% v/v goat serum in PBS) for 1 h at room temperature. After incubation in blocking buffer, cells were incubated overnight at 4 °C with primary antibodies, Orthodenticle homeobox 2 gene (Otx2) (Abgent, AJ1560a 1:200), cone rod homeobox transcription factor(Crx) (Sigma, WH0001406M1) diluted at 1:200 in blocking buffer. After 24 h, cells were washed three times in PBS for 5 min each on a shaking platform at room temperature and incubated at room temperature for up to 1 h with secondary antibodies (Invitrogen’s Alexa Fluor 555 goat anti-rabbit IgG for Otx2 and Alexa Fluor 488 goat anti-rabbit IgG for Crx), pre-diluted in blocking buffer at 1:300.

Secondary antibodies were washed with PBS and cells stained with 4′,6-diamidino-2-phenylindole (DAPI; 1:1000) for 2 min, at room temperature washed in PBS again before mounting in PBS for imaging and storage at 4 °C. A Nikon epifluorescence Eclipse 2000 inverted microscope was used to image the cell markers and analysis was performed using the NIS-elements software version 3.0 (Nikon).

### Quantitative (real-time) PCR (QPCR)

Total RNA was extracted from cultures using RNeasy mini-kit (Qiagen) and reverse transcribed to cDNA using reverse transcriptase PCR (RT-PCR). The amount of cDNA was normalised to glyceraldehyde-3-phosphate dehydrogenase (GAPDH) and β-actin expression. QPCR reactions were performed on a Biorad CFX 96 Connect Real-Time PCR System using the Quantitect SYBR Green PCR Kits (Qiagen) according to the manufacturer’s instructions. The real-time PCR results were analysed using the Biorad CFX Manager 3.0 to calculate normalized relative expression (ΔΔCq) of target genes normalized to relative expression levels of the reference genes β-actin and GAPDH, using the Pfaffl method (Pfaffl 2001). All error bars on QPCR graphs were standard errors of the mean. Primers were from Qiagen: β-actin (QT01680476), GAPDH (QT01192646), P0U5F1-octamer binding transcription factor (OCT-4) (QT00210840), PAX6—paired box 6 (QT00071169), RAX—retina and anterior neural fold homeobox (QT00212667), OTX2 orthodenticle homeobox 2 (QT00213129), SIX3 Six homeobox 3 (QT00211897), CRX Cone-rod homeobox (QT01192632), NRL Neural retina leucine zipper (QT01005165), RHO Rhodopsin (QT00035700).

### Statistical analysis

All error bars represent the standard error of the mean (SEM) and a minimum of 3 replicates were performed for each experiment. Where indicated statistical analyses were performed using the Student’s *t* test or ANOVA for determining the statistical significance of compared data sets. p values <0.05 were considered to be statistically significant.

## Results

The first step towards developing a microwell suspension culture process for the retinal differentiation of human induced pluripotent stem cell (hiPSC) was to control the initial embryoid body (EB) size. The standard manual processes create cellular aggregates by scraping pipette tips along the surface of flasks of attached hiPSC resulting in the formation of a highly heterogeneous mixture of EB shapes and sizes.

**Fig. 1:**
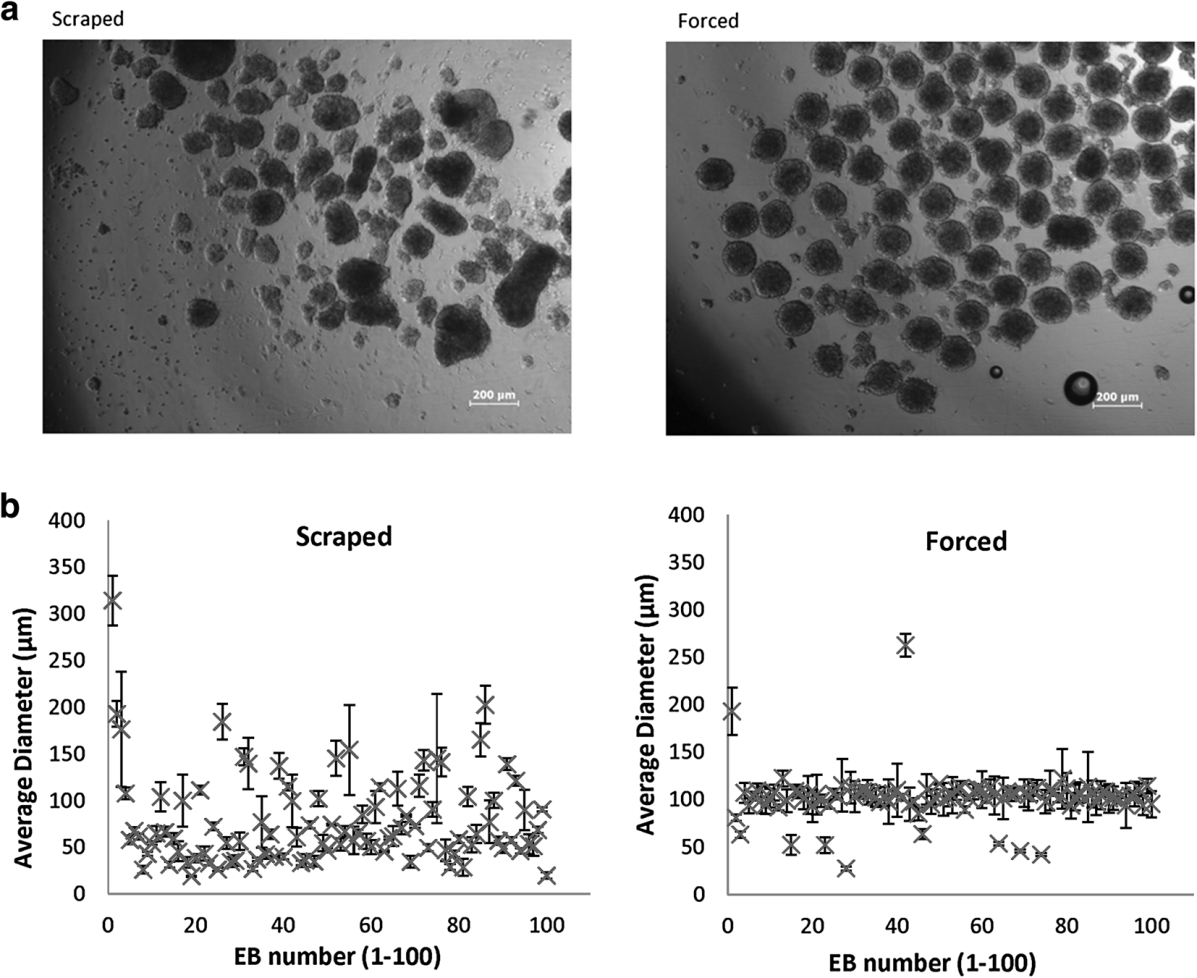
**a** Micrographs of stem cell aggregates formed by scraping and forced aggregation (1000 cells/EB) after 24 h suspension culture. Images were taken at ×4 magnification. **b** Size distribution plots show the variation in size per EB between the scraped and forced aggregation techniques. The average of three measurements per EB (horizontal vertical and diagonal diam. measurements) were taken at 24 h post aggregation as a measure of EB size. *Error bars* represent the standard deviation of the mean for the three measurements per EB

In order to control the EB size, several methods have been developed such as seeding cells in micromass and hanging drops. Hanging drops helped improve EB size reproducibility but was limited to the formation of small EBs (Doetschman et al. 1985; Dang et al. 2002). We used Aggrewell plates which combine the use of microwells with centrifugation to create initial aggregates of 1000 cells per EB (Fig. 1a).

Forced aggregation demonstrated consistent control over EB size in stark contrast to highly heterogeneous scraped EBs (Fig. 1). EBs formed by manual scraping varied greatly in diam. with a broad range between 25–150 µm [mean = 77.6 µm standard deviation (SD) = 48.3] (Fig. 1b). In contrast the mean diam. for EBs formed by forced aggregation was slightly larger (101.4 µm) and far more consistent as reflected by a much lower SD of 24.9. Tighter control over the EB size can be attributed to the precise control over the starting number of input cells per microwells available to form each EB.

**Fig. 2:**
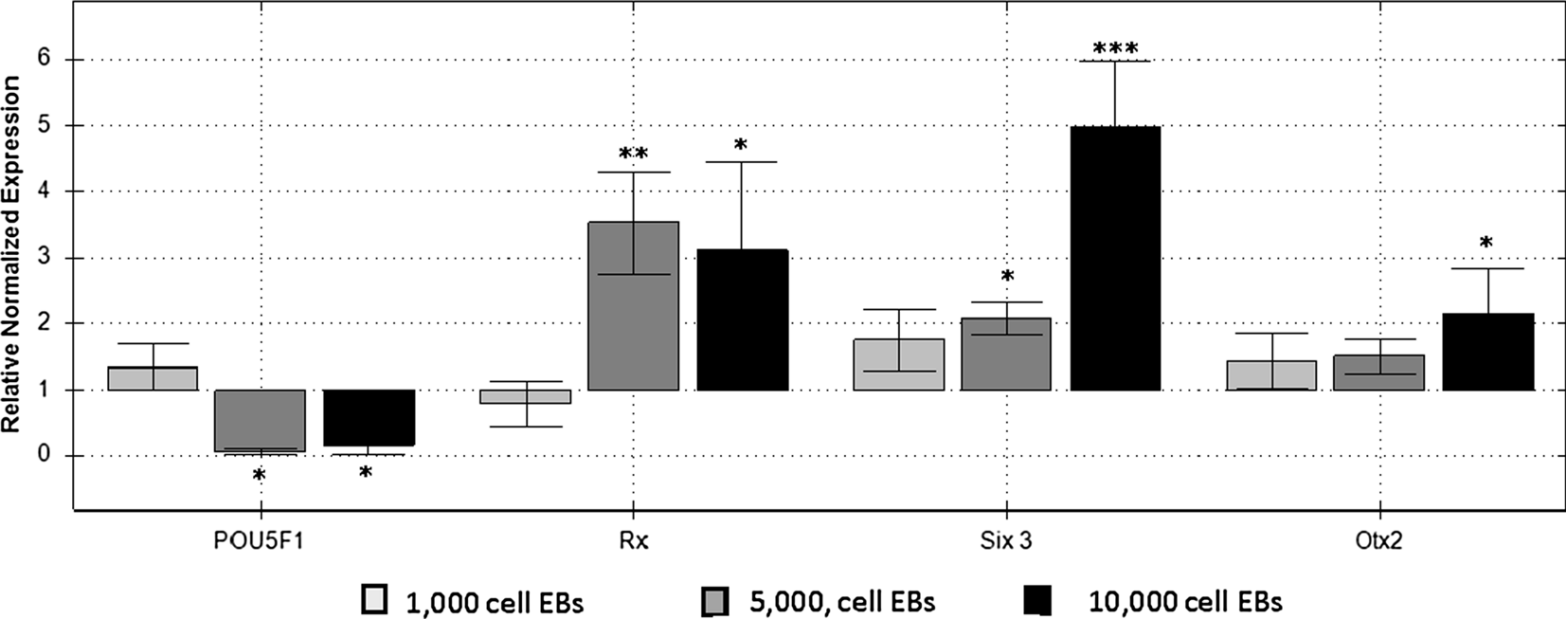
Relative normalized expression of early retinal transcription factor genes, Rx, Six 3 and Otx2 and pluripotency marker P0U5F1 in differentiated EBs at day 3. Samples of EBs made from forced aggregation with 1000 cells/EB, 5000 cells/EB or 10,000 cells/EB cells/EB were normalized against expression profiles from scraped EBs. Each data point represents the mean of three biologically independent replicates (n = 3). One-way ANOVA of gene expression levels were performed against EBs made by scraping (*p < 0.05, **p < 0.01, ***p < 0.001)

In the developing vertebrate embryo, expression of early eye field transcription factors (EFTFs) Rax, Six3 and Otx2 characterise specification of the anterior neural plate, which forms the retina (Bailey et al. 2004). We assessed the impact of EB size on the initial up regulation of EFTFs after 3 days of static suspension culture in retinal differentiation medium (Lamba et al. 2006). Three different EB sizes (1000 cells, 5000 and 10,000 cells/EB) were compared with heterogeneous scraped EBs for the expression of EFTFs analysed by quantitative polymerase chain reaction (QPCR) (Fig. 2).

Out of the three EB sizes evaluated 1000 cells/EB showed comparable gene expression profiles to that of the heterogeneous EBs from scraped control cultures (p > 0.05 for all genes) signifying no improvement in the expression of retinal differentiation potential despite control over EB size. Larger EBs (5000 and 10,000 cells/EB) showed increased expression of Rx, Six3 and Otx2 compared to scraped controls indicating advanced progression towards retinal fates. The 5000 cell EBs displayed a 3.52-fold (p < 0.01) increase in expression of Rx and a 2 fold up-regulation of Six3 (p < 0.05) compared to scraped controls. EBs composed of 10,000 cells also showed significant up-regulation of Rx (3.12-fold, p < 0.05) and Six3 (5 fold, p < 0.001) compared to the scraped controls. The 5000 and 10,000 cell EBs demonstrated input EB size can influence retinal differentiation. It has been shown that changing the EB size increased the yield of endothelial cells by a 2 fold (Moon et al. 2014). Moreover, larger EBs contain neural cells (Nakano et al. 2012) and the generation of retinal progenitor cells from hESC was optimised upon the usage of and EB of 9 K cells/EB (Bauwens et al. 2008).

In agreement with the work of others, EB populations from both scraped and forced aggregation co-expressed the pluripotency marker P0U5F1 alongside the early markers for retinal differentiation at day 3 (Mellough et al. 2012). Relative normalised expression of P0U5F1 in the 1000 cell EBs was also indistinguishable from that of the scraped controls (p > 0.05). In contrast the 5000 cell EBs showed a 16.6-fold (p < 0.05) reduction in P0U5F1 expression while the 10,000 cell EBs exhibited a 6.3-fold downregulation (p < 0.05) of the same marker when compared with scraped controls. The increased expression of EFTFs combined with concurrent down-regulation of P0U5F1 in the 5000 and 10,000 cell EBs support the hypothesis that larger EBs were differentiating toward retinal fates at an accelerated pace as compared with scraped controls.

To establish a permissible orbital shaking frequency for shaken culture, 30-40 EBs formed of 5000 and 10,000 cells were maintained in orbital shaking suspension in ultra-low attachment 24 well plates at a series of different shaking frequencies, between 50 and 240 rpm. Over 7 days of culture EBs were assessed by microscopy for survival as separate intact entities, agglomeration or dissolution into the medium. Typical micrographs of EBs after 7 days in culture at different shaking speeds show low speeds (50 rpm) caused EB agglomeration into large clumps; while speeds of 150 rpm resulted in EB dissolution into the medium (Supplementary Fig. 1). At a shaking speed of 120 rpm, EBs survived as individual intact entities throughout the 7 days culture period.

**Fig. 3:**
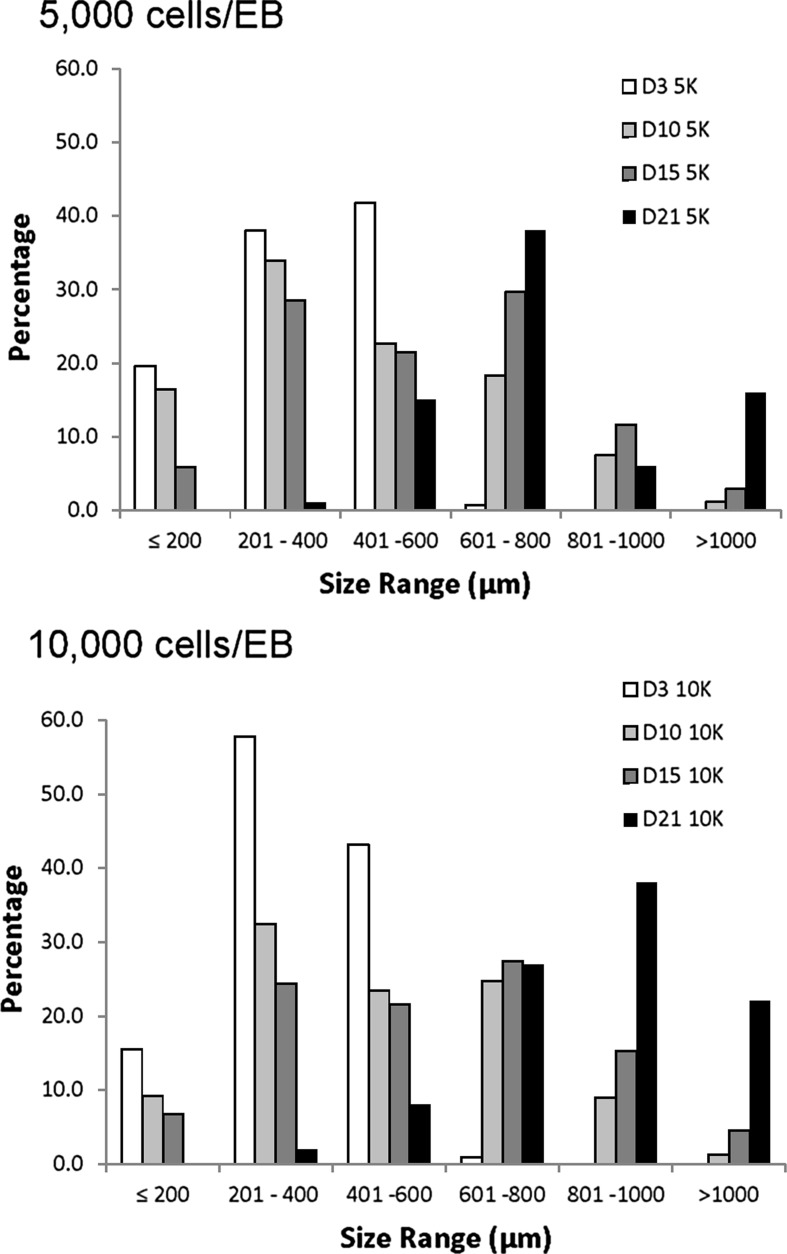
Frequency distribution plot of EB size variation for 5000 and 10,000 cells/EB. Average EB diam.s were measured at days 3, 7, 10 and 15 from triplicate readings per EB for n = 100 EBs at each time point. The represented EB size distribution shows absolute frequencies of numbers of EBs measured and categorised into each size bracket in 50 µm increments between ≤50 and ≤1200 µm for each time point. EBs were imaged using a phase contrast microscope and average diameters measured using Image J software

We next assessed how larger EBs (5000 and 10,000 cells/EB) expanded during a longer culture period of 15 days (Fig. 3). These results shows that EBs expand in culture forming a new distinct population of large EBs over the size of 500 µm. This occurs at both 5000 and 10,000 cells/EB as does the persistence of a significant population of smaller EBs. Controlling the EB starting size and the implementation of mixed conditions was not sufficient to minimise the formation of a very wide range of aggregate sizes in agreement with previous studies using stirred tank bioreactors (Miranda et al. 2015). Changing the EB size can affect O_2_ diffusion, availability of soluble factors and the nutrient mass transfer rates. The internal O_2_ concentration varies between different size EBs. Moreover, EBs with 200 µm diam. have 50% higher O_2_ concentration at their core when compared with larger 400 µm diam. EBs (Van Winkle et al. 2012).

Both 5000 and 10,000 cells/EB cultures displayed morphological features indicative of retinal differentiation (Supplementary Fig. 2).

EBs from both input size populations displayed similar mixtures of dense, dark centred EBs (features indicated by arrows iii and v in Supplementary Fig. 2) (Mellough et al. 2012). Morphological signs of neural epithelial induction, were also detected with the appearance of secondary structures in both cultures (indicated by arrows i and ii in Supplementary Fig. 2), occasional invaginations along their edges (arrow iii) and optic cup like shapes (arrows iv and v) consistent with the published literature.

**Fig. 4:**
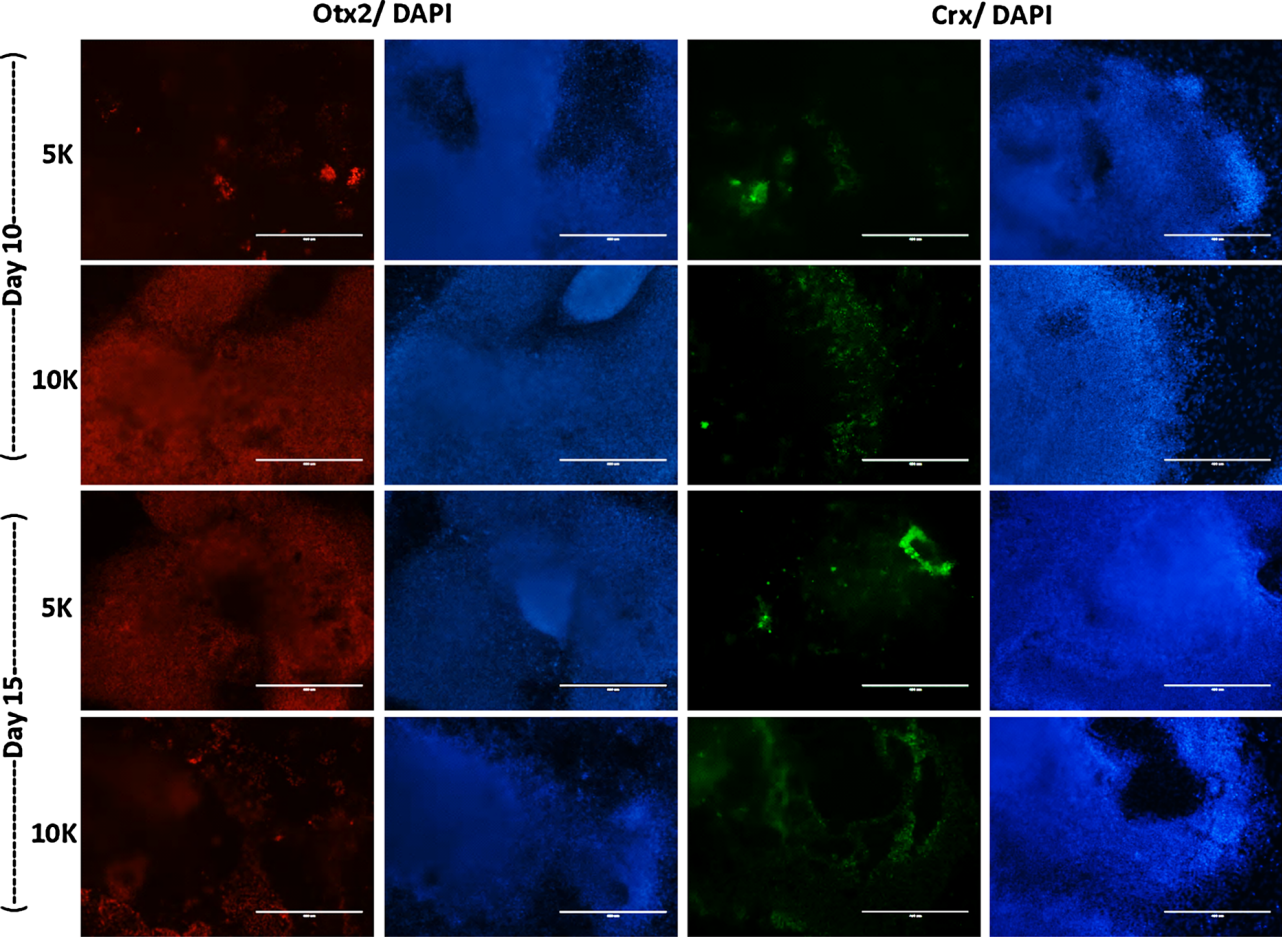

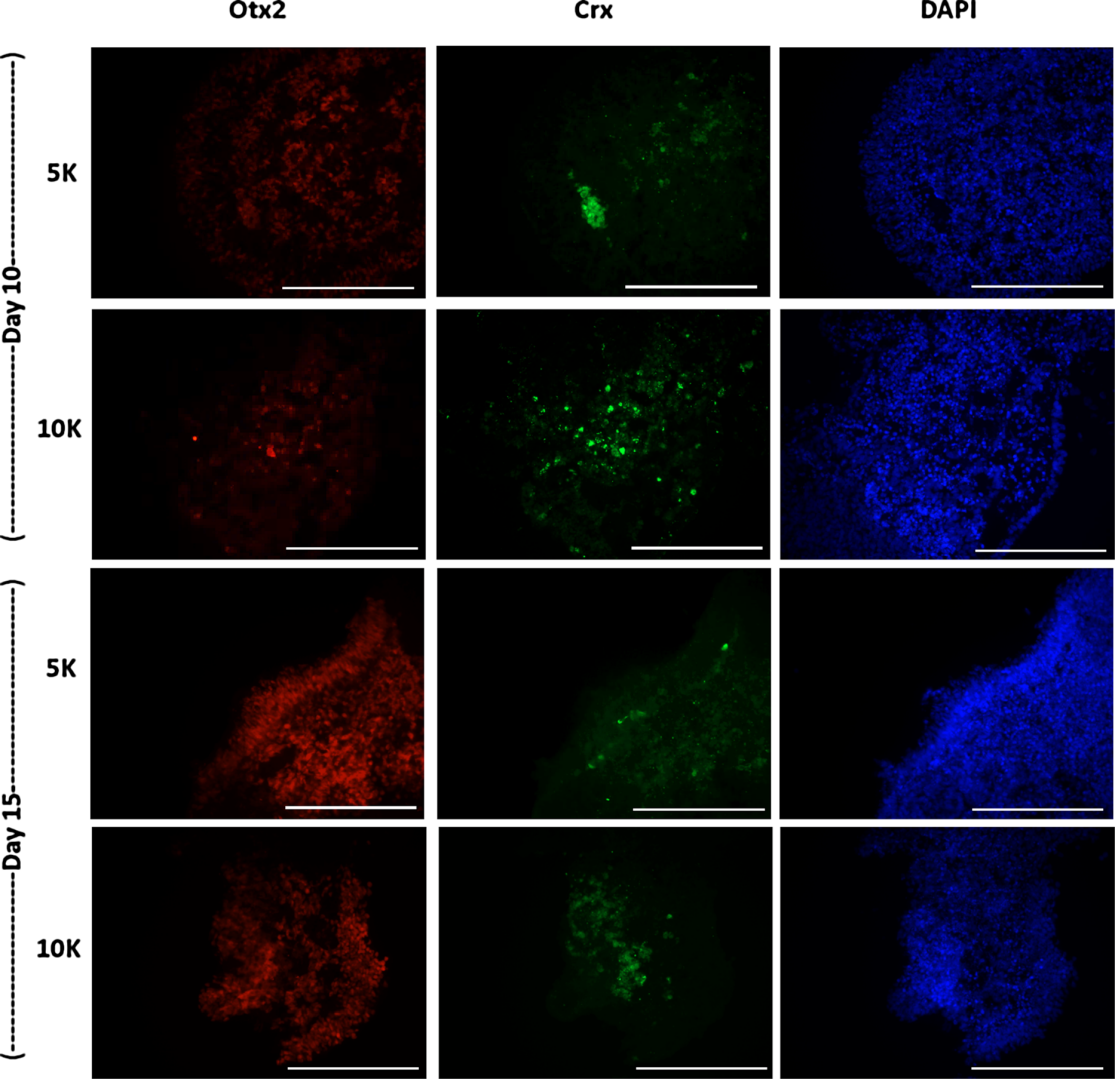
Immunocytochemistry of **a** adherent and **b** cross-sections of orbital shaken retinal EBs. EBs made from 5 K or 10 K cells at days 10 and 15, were stained for early eye field transcription factor Otx2, photoreceptor precursor marker Crx and nuclear stain DAPI. *Scale bars* 400 µm

EBs of both sized were cryosectioned and stained for an early (Otx2) and later (Crx) marker of the retinal differentiation process (Fig. 4). These results showed the expression of both markers in all of the shaken microwell cultures. Expression levels were comparable with attached controls.

**Fig. 5:**
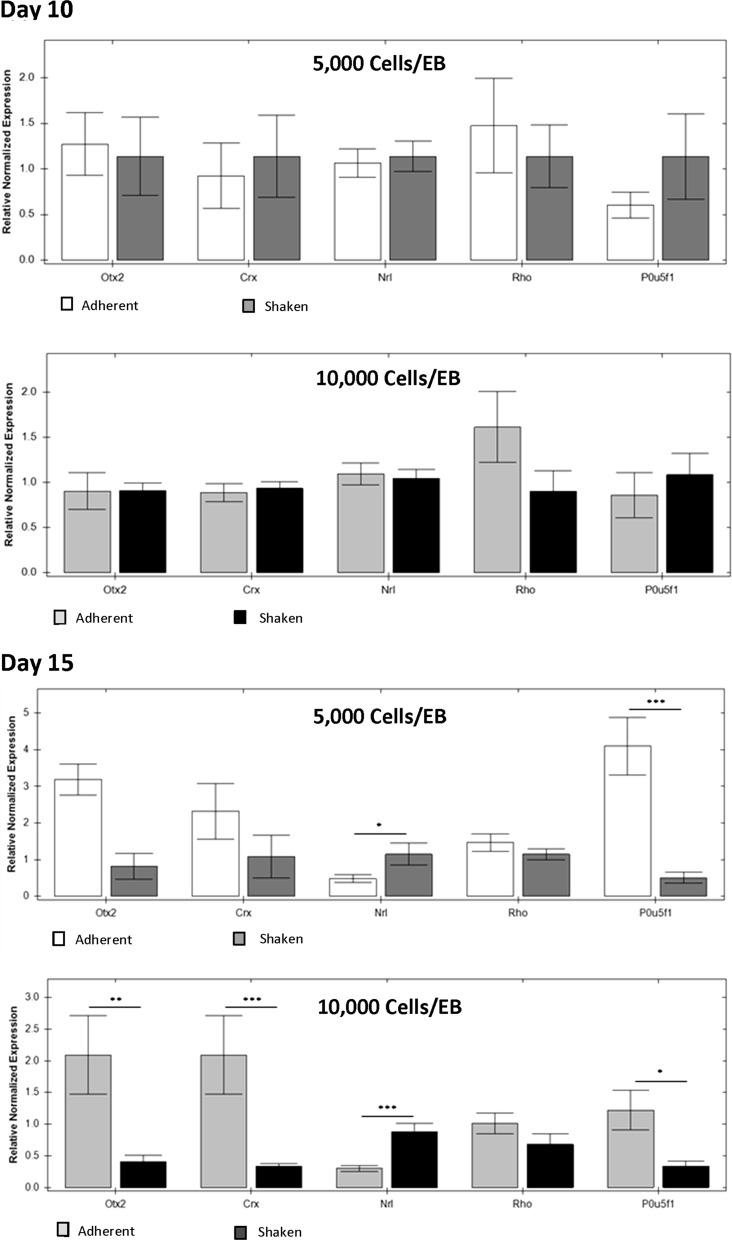
RT-PCR analysis of Otx2, Crx, Nrl, Rho and P0U5F1gene expression profiles of EBs at day 10 and day 15 of shaken retinal differentiation culture initiated with EBs formed of (i) 5000 or (ii) 10,000 cells/EBs, normalized relative to size-matched adherent control cultures. Each data point represents the mean of three biologically independent replicates (n = 3). One-way ANOVA of gene expression levels were performed against EBs made by scraping (*p < 0.05, **p < 0.01, ***p < 0.001)

To provide quantitative support for the morphological and immunohistochemical evidence of retinal differentiation, QPCR analysis quantified the variation in expression of retinal gene markers. The impact of shaking culture on retinal gene expression was observed by normalizing against retinal gene expression of size-matched adherent EB cultures (Fig. 5).

After 10 days of shaken culture there were no significant differences (p > 0.05) between the adherent and shaken cultures in the expression of Otx2, Crx, Nrl, rhodopsin or P0U5F1. By day 15 there were no significant differences in the expression of Rhodopsin or Crx at 5000 cells/EB. Shaking culture resulted in an up regulation of Nrl expression at both sizes, however this came at the expense of Crx for the larger 10,000 cells/EB condition. Under both conditions shaking resulted in the down regulation of P0U5F1 indicating that mixed conditions may reduce the persistence of pluripotent cells.

Size-matched EBs (5000 cells/EB) day 15, showed comparable levels of relative normalised expression of early retinal markers Otx2 (p > 0.05) and photoreceptor precursor marker Crx (p > 0.05) in adherent and orbital shaken cultures. In contrast, expression of later photoreceptor-specific marker Nrl was 2.39-fold (p < 0.05) higher in orbital shaken cultures than adherent controls showing an advantage of using shaken culture to produce Nrl positive cells which are required for maximising the integration capacity of mammalian photoreceptor precursor cells (West et al. 2012).

## Discussion

Since the first derivation of human embryonic stem cells (hESCs) and subsequent discovery of human induced pluripotent stem cells (hiPSCs) there have been international efforts to derive differentiated cell populations for both clinical, screening and scientific applications.

The vast majority of these studies are undertaken in standard cell culture laboratories where static culture of cells is the norm. Although scalable, better controlled and more homogenous cell culture protocols can be achieved in stirred tank bioreactors many labs do not have the expertise or equipment to undertake this transition. A number of groups have pioneered the development scale-down suspension bioreactors such as microwells originally designed to mimic large-scale bioreactors for process development studies. They represent a simple and cost effective route to evaluate whether static stem cell culture processes can be transferred into suspension bioreactor formats. A major advantage of the technology is that the only major capital expenditure for standard cell culture laboratories is a relatively cheap shaking platform. Stem cell culture processes contain expensive growth factors during both the expansion and differentiation steps meaning that a typical academic stem cell laboratory may find the cost of even a single 100 ml bioreactor run prohibitively high. Shaking microwells used in this study are 3.4 ml with a working volume of 0.5 ml meaning that the medium usage is equivalent to standard adherent cultures.

In this report we adapted a widely used adherent protocol for the pluripotent stem cell retinal differentiation (Lamba et al. 2006) to shaking microwells in two steps. Firstly, using commercially available Aggrewell plates, to identify the optimum starting size for the embryoid bodies (EBs).

Once inoculated into the shaking microwell environment we were able to identify a permissive shaking frequency (120 rpm) which did not destroy the cellular aggregates resulting in the expansion of EBs with time. It is possible that optimum shaking speeds are affected by EB size. However, the resulting EBs expressed key markers associated with retinal differentiation at similar levels to the adherent protocol. These studies demonstrate that shaking microwells are a suitable proof-of-concept system, enabling stem cell laboratories to quickly and cheaply assess whether their static cell culture processes can be adapted to a mixed suspension format.

Given that the relationship between shaking microwells and stirred tank bioreactors is well established, experiments in this format will give many groups the confidence before investing significant time and effort in more sophisticated rigs and large amounts of media.

Orbital shaking platforms have been applied to the spontaneous (Sargent et al. 2010; Kinney et al. 2012), endodermal (Carpenedo et al. 2007) and cardiomyocytes (Niebruegge et al. 2009) differentiation of embryonic stem cells (ESC). These studies in general used much lower shaking speeds (25–65 rpm). Using hiPSC we found that these low speeds resulted in significant clumping. There are many differences between the different systems which may account for the discrepancy including the size of the culture chamber, the shaker platform’s orbit, cell type, EB formation technique, media composition, donor and embryonic versus iPSC derivation. Importantly, both EB starting size and shaking speed are two critical variables which must be investigated during the early adoption of shaking cell culture processes.

In the experiments described here we have adapted a widely used protocol for early retinal differentiation to the shaken microwell format. The combination of EB size control with orbital shaken culture is not only suitable for initiating retinal differentiation but also eliminates the requirement for the murine Engelbreth-Holm-Swarm tumour derived substrate, Matrigel used frequently in retinal differentiation protocols (Lamba et al. 2006; Eiraku et al. 2011; Nakano et al. 2012; Zhong et al. 2014). Whilst Matrigel provides a niche for growth factor binding and mechanical support for adherent EB cultures, it contains a mix of ingredients in undefined quantities and is therefore a source of variability, debatably unsuitable for protocols aiming to derive cells for clinical use.

Elsewhere, static suspension culture methods have been used to differentiate mouse embryonic stem cell (mESC) into self-forming optic cups, as developed by the Sasai laboratory (Eiraku et al. 2011). The same approach was also successful in producing human ESC derived self-forming optic cups (Nakano et al. 2012) and hiPSC initiated optic cup derived functional photoreceptors (Zhong et al. 2014). These experiments were all carried out under static conditions and may benefit from the introduction of orbital shaken culture to improve scalability and reproducibility.

One drawback of shaking microwells is that they do not include many potentially beneficial functionalities associated with suspension bioreactors such as pH, O_2_ control and medium feeding regimes. The elimination of medium exchanges and the potential to implement fed batch and perfusion regimes eliminate the deleterious effects associated with regular medium exchanges (Veraitch et al. 2008). In the case of the retinal differentiation process we envisage that further gains in the yield and efficiency of the process can be realised through the application of more advanced bioreactors. These systems are available at a wide range of scales from 10 ml up to 20,000 l. Therefore, this proof of concept study in shaking microwells opens the door to a wide range of production scales suitable for the production of cells for a wide range of applications.

## Electronic supplementary material

Below is the link to the electronic supplementary material.

Supplementary material 1 (DOCX 2506 kb)
