# Supplementary material for: Early retinal differentiation of human pluripotent stem cells in microwell suspension cultures
Source: Biotechnol Lett. 2016 Nov 3;39(2):339–50. doi: 10.1007/s10529-016-2244-7 (PMC5247545; doi:10.1007/s10529-016-2244-7)
Supplement: Supplementary file 1 — Supplementary material 1 (DOCX 2506 kb) [file 10529_2016_2244_MOESM1_ESM.docx]

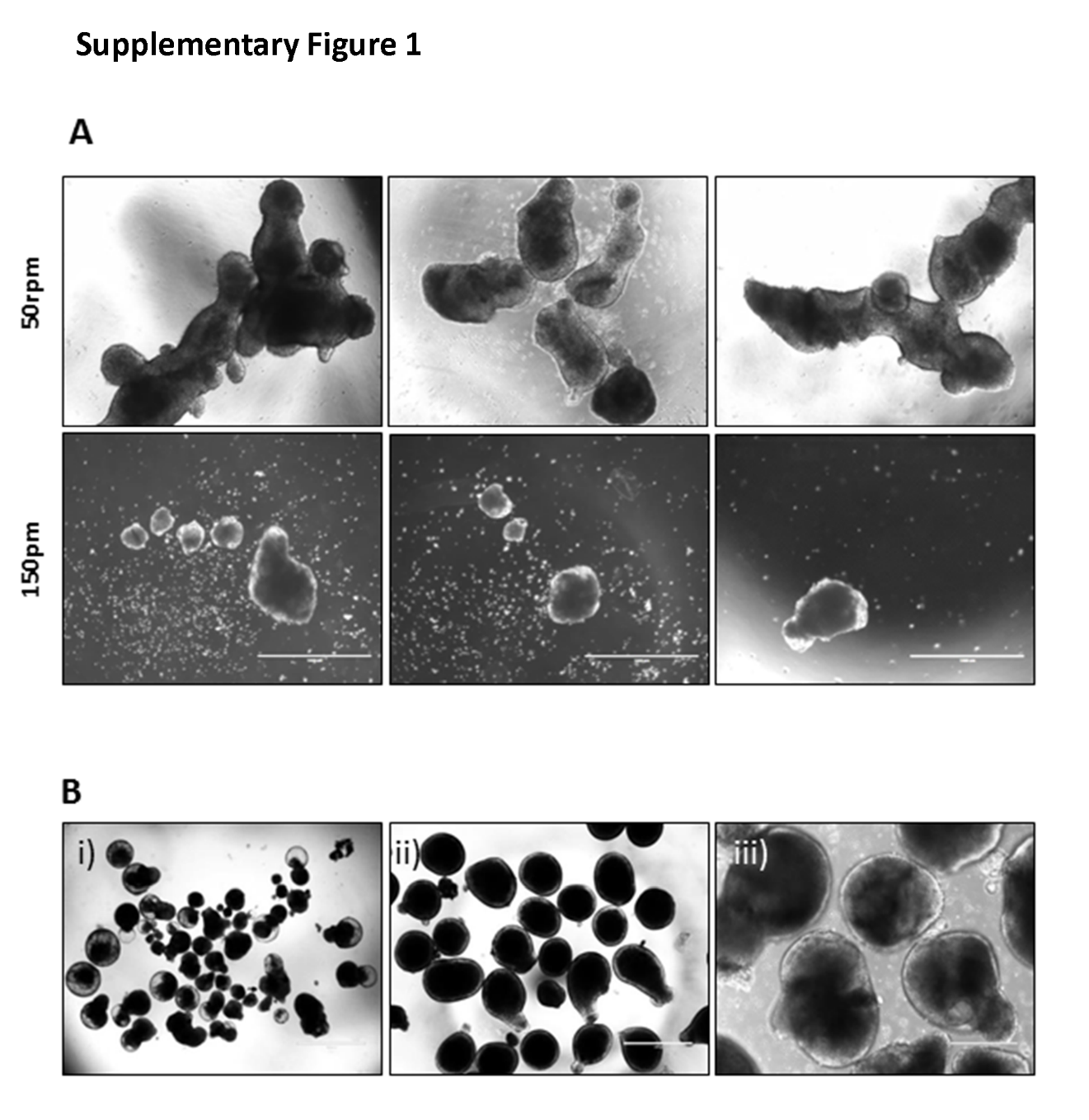


**Supplementary Figure 1 (a)** Representative micrographs of stem cell aggregates (40-50 10K EBs per condition) cultured in retinal differentiation medium in rotary suspension on day 7 at different shaking speeds: 50rpm (ai) and 150rpm (aii). (b) shows improved survival of EBs made from 10K cells at the optimum shaking speed of 120rpm at different magnifications 4x (bi), 10x (bii) and 20x (biii). All images were taken using a Nikon Phase and Fluorescence Microscope. Scale bars = 100µm.


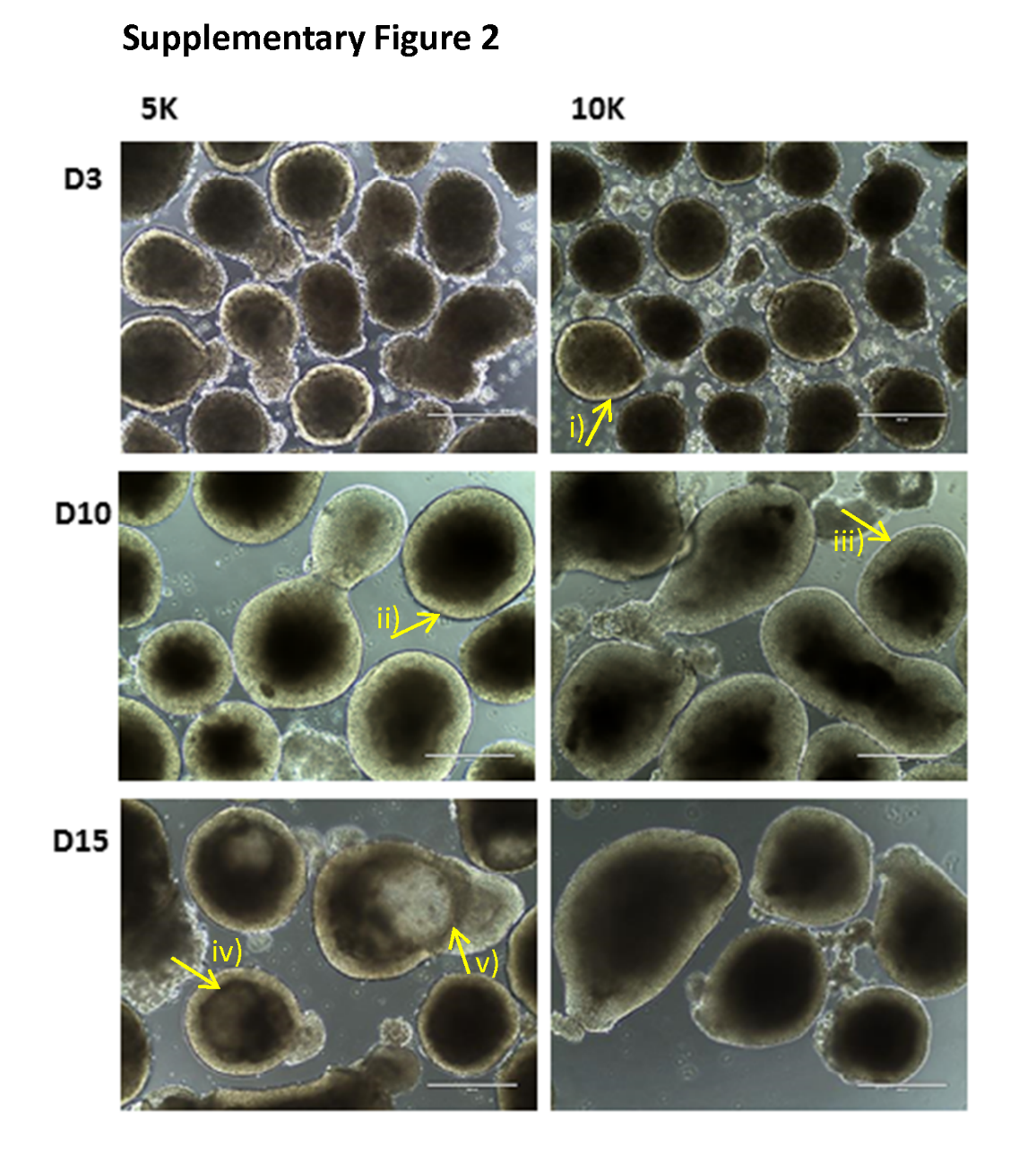


**Supplementary Figure 2** Representative micrographs of MSU001 retinal EBs made from 5000 or 10,000 cells/EB at days 3, 10, and 15 of retinal differentiation. Yellow arrows i to v highlight appearance of secondary structures in cultures from phase dark rings around EB perimeters, occasional invaginations along their edges (arrow iii) and optic cup like shapes (arrows iv and v) Images are at 10x magnification and scale bars = 400µm.
